# The Identification of a Quantative Trait Loci-Allele System of Antixenosis against the Common Cutworm (*Spodoptera litura* Fabricius) at the Seedling Stage in the Chinese Soybean Landrace Population

**DOI:** 10.3390/ijms242216089

**Published:** 2023-11-08

**Authors:** Lin Pan, Junyi Gai, Guangnan Xing

**Affiliations:** Soybean Research Institute & MARA National Center for Soybean Improvement & MARA Key Laboratory of Biology and Genetic Improvement of Soybean & State Key Laboratory of Crop Genetics, Germplasm Enhancement and Utilization & State Innovation Platform for Industry-Education Integration in Soybean Bio-Breeding & Jiangsu Collaborative Innovation Center for Modern Crop Production, Nanjing Agricultural University, Nanjing 210095, China

**Keywords:** soybean (*Glycine max* (L.) Merr.), antixenosis, common cutworm (*Spodoptera litura* Fabricius), landrace, restricted two-stage multi-locus genome-wide association study (RTM-GWAS), QTL-allele matrix, recombination potential, gene function annotation

## Abstract

Common cutworm (CCW) is an omnivorous insect causing severe yield losses in soybean crops. The seedling-stage mini-tray identification system with the damaged leaf percentage (DLP) as an indicator was used to evaluate antixenosis against CCW in the Chinese soybean landrace population (CSLRP) under three environments. Using the innovative restricted two-stage multi-locus genome-wide association study procedure (RTM-GWAS), 86 DLP QTLs with 243 alleles (2–11/QTL) were identified, including 66 main-effect loci with 203 alleles and 57 QTL-environment interaction loci with 172 alleles. Among the main-effect loci, 12 large-contribution loci (*R*^2^ ≥ 1%) explained 25.45% of the phenotypic variation (PV), and 54 small-contribution loci (*R*^2^ < 1%) explained 16.55% of the PV. This indicates that the CSLRP can be characterized with a DLP QTL-allele system complex that has not been found before, except for a few individual QTLs without alleles involved. From the DLP QTL-allele matrix, the recombination potentials expressed in the 25th percentile of the DLP of all possible crosses were predicted to be reduced by 41.5% as the maximum improvement and 14.2% as the maximum transgression, indicating great breeding potential in the antixenosis of the CSLRP. From the QTLs, 62 candidate genes were annotated, which were involved in eight biological function categories as a gene network of the DLP. Changing from susceptible to moderate plus resistant varieties in the CSLRP, 26 QTLs had 32 alleles involved, in which 19 genes were annotated from 25 QTL-alleles, including eight increased negative alleles on seven loci and 11 decreased positive alleles on 11 loci, showing the major genetic constitution changes for the antixenosis enhancement at the seedling stage in the CSLRP.

## 1. Introduction

Soybean is one of the most important crops in the world because of its high contents of plant protein and oil. The common cutworm (CCW, *Spodoptera litura* Fabricius) is a major leaf-feeding pest, characterized by an omnivorous diet, overeating and fast fecundity [1,2]. This insect is widely disseminated in tropical and subtropical regions such as Asia, Africa, North America and Oceania [3,4]. Cardona et al. [5] reported that if the damage of the CCW is not controlled, crop yields would be reduced by 50–100%. Until now, the major method for controlling pests for field crops has depended on pesticide application, which is costly and environmentally unfriendly [6]. The development of varieties that are resistant to CCW has been a sustainable and environmentally friendly method within pest management systems due to its effectiveness in controlling pests and reducing the use of insecticides [7,8].

Host plant resistance can be distinguished into three categories: antibiosis, antixenosis or non-preference and tolerance [9]. The research on antixenosis is relatively lacking due to fact that the previous methods of evaluating antixenosis against pests using adult plants under field or net-room conditions were time-consuming, labor-intensive, and lacking precision [2]. Generally, there are three approaches for the identification of insect resistance, i.e., identification with natural insect sources in the field [10,11], identification in a net room with artificial infestation [2,12] and biological identification in a laboratory [13,14]. Among them, the former two are the major ones for antixenosis, with the DLP [2,15,16], the preference for oviposition [17] and the larval density [11] as indicators. For an efficient and high-throughput evaluation of antixenosis, Xing et al. [2] proposed a seedling-stage mini-tray identification system in greenhouses using the damaged leaf percentage (DLP) as an indicator, whereas the often-used indicators for antibiosis (also known as bioassays) are the larval weight, pupal weight and duration of the larval stage [18,19,20].

Breeding progress depends on potential germplasm resources, such as landraces. The key for the utilization of the required genes in the germplasm population is to explore the genes/QTLs with their superior alleles. Soybean originated in China, where the crop has been cultivated for more than 5000 years [21]. Over the course of a long history, ancient Chinese farmers developed a great number of landraces, which accumulated tremendous genetic variation and, therefore, are the most important genetic reservoir for pest-resistance breeding [22].

Molecular marker technology has helped to find resistance QTLs with elite alleles against CCW. For antibiosis, two major antibiosis QTLs (*CCW-1* and *CCW-2*) against the CCW were identified on chromosome 7 [8]. For antixenosis, Oki et al. [23] identified two QTLs, *qRslx1* and *qRslx2*, on chromosomes 7 and 12, respectively, via linkage mapping using a comparison between the test line and a standard variety. Oki et al. [24] identified a further two QTLs (*qRslx4* and *qRslx3*) on chromosomes 2 and 7 for antixenosis against CCW, using an RIL population derived from a cross between wild soybean (*Glycine soja*) and ‘Fukuyutaka’ via linkage mapping. Kim et al. [12] revealed *qCCW6-1*, *qCCW10-1* and *qCCW12-2* for antibiosis against CCW using the larva weight and pupa weight as indicators, and *qCCW10-1*, *qCCW10-2* and *qCCW12-1* for antixenosis against CCW using the DLP as an indicator in two RIL populations of NJRIKY and NJRIXG via linkage mapping.

The above results were mainly obtained from a few bi-parental populations. However, insect resistance in germplasm and breeding populations may be a complex quantitative trait, involving a large number of QTLs/genes with their multiple alleles. Linkage mapping based on bi-parental populations could detect two alleles only on each locus, which did not fit a breeding population with multiple alleles [25]. With the development of high-throughput genomic sequencing technologies, genome-wide association studies (GWASs) for natural populations have been widely used in dissecting QTLs with alleles of complex traits [26]. Although GWASs have been used for insect resistance [27], few have been used for CCW resistance (especially antixenosis) in soybean crops. Kim et al. [6] detected 26 and 43 QTLs of antibiosis to CCW in 2009 and 2011, respectively, in a Chinese soybean germplasm population, whereas Wang et al. [28] detected six, three and nineteen QTLs using the larva weight, pupa weight and larva feeding leaf weight as resistance indicators, respectively, in a germplasm population.

The most popular GWAS procedure that has been previously used is the mixed linear model (MLM) procedure [29]. But the MLM-GWAS procedure based on SNP markers can only detect two alleles at a locus, which is useful for finding some major QTLs but not for detecting the multi-allelic variation hidden within the germplasm population. Indeed, plant breeders are more interested in operating whole QTL-allele systems of traits in breeding programs. In addition, the previous GWAS methods, based on single-locus models, may lead to tremendous false positives due to interference from neighboring loci. To avoid the above problems, an innovative restricted two-stage multi-locus model, GWAS (RTM-GWAS), was developed [30]. First, the RTM-GWAS method matches the loci with multiple alleles by constructing SNP linkage disequilibrium block (SNPLDB) markers with multiple haplotypes. Second, the RTM-GWAS method combines the multi-locus model and heritability control for total genetic contribution to reduce both false positives and false negatives. Thirdly, the RTM-GWAS procedure uses two-stage analysis combined with an experimental design to reduce calculation and raise precision. The RTM-GWAS procedure has recently been applied to identify QTL-allele systems for seed isoflavone content [31] and seed oil content [32] in soybean germplasm and breeding populations.

From the above, the present study aimed at (i) evaluating antixenosis against the common cutworm at the seedling stage using a high-throughput phenotyping procedure proposed by Xing et al. [2] in a Chinese soybean landrace population (CSLRP); (ii) exploring the consistency of antixenosis as well as antibiosis between the seedling and adult stage and choosing the best resistance sources; (iii) identifying the whole antixenosis QTL-allele system and its differentiation among different degrees of antixenosis in the CSLRP using RTM-GWAS; (iv) predicting the genetic potentials of the CSLRP through recombination among the accessions; and (v) predicting the candidate gene system of antixenosis of soybeans to common cutworms.

## 2. Results

### 2.1. Antixenosis Variation in CSLRP

The DLP value averaged over three years for antixenosis to CCW showed a wide variation, ranging from 17.2% to 79.9%, with an overall mean of 44.5%. Its heritability was estimated as 77.3%, with a genetic coefficient of variation (*GCV*) of 24.1% (Table 1). The ANOVA over three environments indicated significant differences among genotypes and genotype-by-environment interactions (Table S1). The *F*-values in Table 1 also show significant phenotypic variation among and within the six ecoregions, indicating abundant variation in antixenosis among and within ecoregions. The varieties from ecoregions II, V and VI had relatively high antixenosis against CCW compared to those from ecoregions III and IV, while ecoregion I was between the two sets (Table 1). Ten varieties (S01–S10) with high antixenosis (low DLP) and five highly susceptible (high DLP) varieties (S11–S15) were selected and used in subsequent evaluation for their antixenosis and antibiosis at the adult stage. Their names are listed in the notes of Table S2.

**Figure 1:**
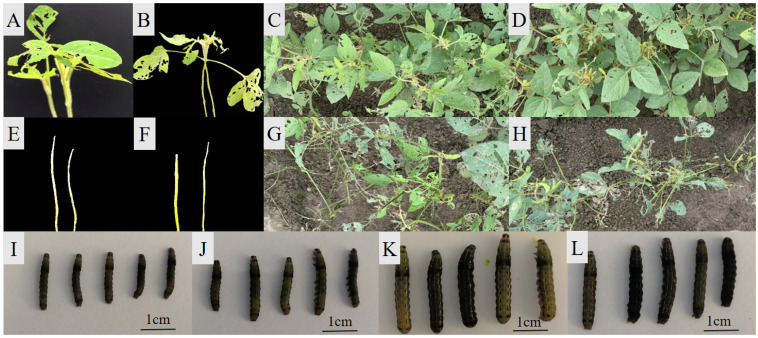
The performance of antixenosis and antibiosis at seedling and adult stages on highly resistant and susceptible accessions. (**A**–**D**): Highly resistant accession S07 and Lamar at seedling (**A**,**B**) and adult stages (**C**,**D**), respectively. (**E**–**H**): Highly susceptible accession S13 and NN89-29 at seedling (**E**,**F**) and adult stages (**G**,**H**), respectively. (**I**–**L**): larvae after 12 days of feeding on highly resistant accession S06 and Lamar (**I**,**J**) and highly susceptible accession S13 and NN89-29 at seedling stage (**K**,**L**), respectively.

There was a significant difference in antixenosis at the adult stage between the highly resistant varieties and highly susceptible varieties at the seedling stage, with the performance of antixenosis to CCW basically consistent for the two sets of selected accessions (Table S2). Five accessions (S02, S05, S06, S07 and S08) with high antixenosis and antibiosis at the adult stage were better than or equal to the resistant control Lamar (Figure 1, Table S2). The positive correlation among all indicators of antibiosis and antixenosis at the seedling and adult stage reached a high degree at a very significant level (Table S3).

### 2.2. QTL-Allele System of Antixenosis in CSLRP

**Figure 2:**
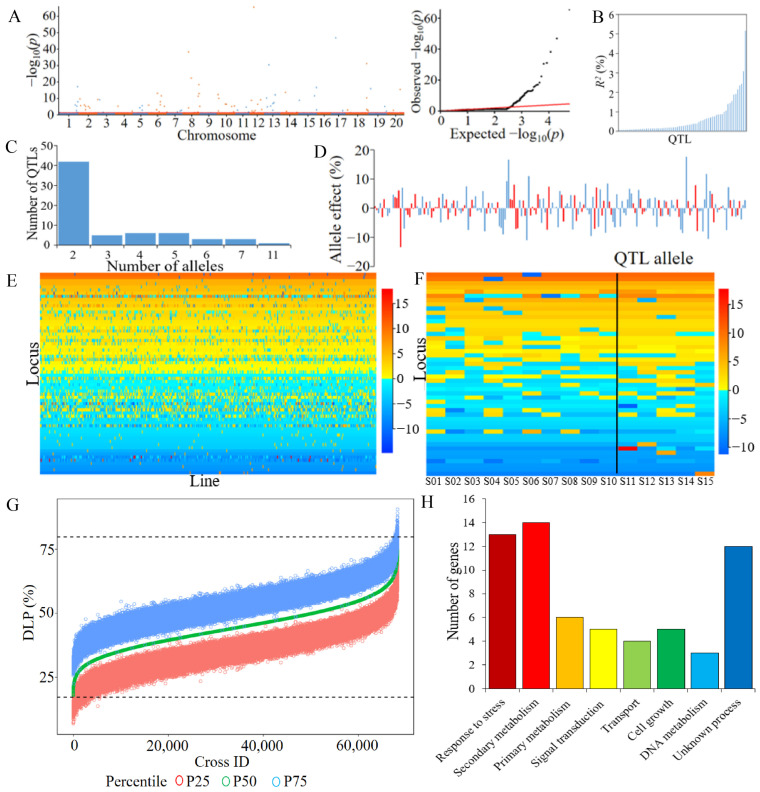
Identification of DLP QTL-allele system using RTM-GWAS. (**A**): Manhattan (**left**) and quantile-quantile plot (**right**). The vertical axis indicates the −log_10_*p* value, and the horizontal axis represents chromosomes, with the solid red line indicating the genome-wide threshold of *p* = 0.05. Blue and orange are used to distinguish the QTLs of adjacent chromosomes. (**B**): Phenotypic contribution of the detected 66 DLP QTLs. (**C**): Distribution of number of alleles on the 66 DLP QTLs. (**D**): DLP allele effects of the 66 main-effect QTLs. Red and blue are used to distinguish the effect of adjacent QTL alleles. (**E**): DLP QTL-allele matrix. The horizontal axis represents variety in CSLRP, while the vertical axis denotes the detected QTLs on chromosomes. The allele effects are expressed in colors, where the red color indicates a positive value and blue color indicates a negative value, with dark color indicating effect sizes. (**F**): The QTL-allele matrix of 66 main-effect QTLs in the selected 10 highly resistant and 5 highly susceptible accessions. (**G**): The distribution of predicted progeny DLP of possible crosses in CSLRP. The horizontal axis represents the possible crosses arranged in an increasing order of the predicted 50th percentile DLP, while the vertical axis indicates the predicted DLP with P25, P50 and P75 or the 25th, 50th and 75th percentile of each cross expressed in different colors. The dotted lines indicate the maximum and minimum of the observed accessions in CSLRP. (**H**): GO biological process distribution of candidate genes annotated from the 86 antixenosis QTLs against CCW in CSLRP.

At the first step of RTM-GWAS, 14,703 SNPLDBs out of 29,234 were preselected, from which 86 DLP QTLs for antixenosis against CCW in CSLRP were identified through stepwise regression with forward selection and backward deletion at the second step. Among the DLP QTLs, 66 were significant main-effect QTLs, 57 were significant QTL× environment interaction (QEI) QTLs and 37 were involved in both main and interaction effects (Figure 2A, Table 2). The 86 DLP QTLs were distributed on 18 soybean chromosomes, except the 9th and 16th chromosomes, which each had 2–8 QTLs, while chromosomes 6, 8, 12 and 13 harbored the main-effect QTLs more than the others. For main-effect QTLs, their phenotypic contribution ranged from 0.06% to 5.16% each, with 12 large-contribution (LC) QTLs, each with *R*^2^ ≥ 1%, explaining 25.45% phenotypic variation (PV), while 54 small-contribution (SC) QTLs each had *R*^2^ < 1%, explaining a total of 16.55% PV, in a total of 42.00% PV. Similarly, the 57 significant QEI QTLs explained 15.85% PV, in which 1 LC QEI QTL (*R*^2^ ≥ 1%) explained 1.06% PV and 56 SC QEI QTLs explained 14.79% PV. Altogether, 66 main-effect plus 57 QEI QTLs contributed 57.85% PV. Since the QEI QTLs had a relatively small contribution to PV compared to the main-effect QTLs, the subsequent analysis will focus on the latter.

The *q-DLP-12-4* on chromosome 12 contributed 5.16% to PV, the highest contribution among the detected QTLs, while the second and third highest contributions were by *q-DLP-15-2* (3.08%) and *q-DLP-18-4* (2.44%), respectively (Table 2). There were 24 QTLs that had more than two alleles, while the other 42 QTLs contained only two alleles among the 66 main-effect QTLs, in a total of 203 alleles, including 99 positive- and 104 negative-effect alleles. The allele number per QTL ranged from 2 to 11, with an average of 3.1, and the QTL *q-DLP-13-2* had 11 alleles, the largest number of alleles per locus (Table 2). The allele effects ranged from 0.01 to 17.6 for positive-effect alleles and −13.4 to −0.14 for negative-effect alleles (Figure 2D). Approximately 92.6% of allele effects were between −8.0 and 8.0, indicating that alleles with extremely high or low phenotypic effects were not common. The 66 significant main-effect QTLs with 203 alleles can be organized into a QTL-allele matrix (66 loci × 370 accessions), which represents the total genetic structure of the population as well as that of each accession. It can be seen that the phenotypic differences among accessions depend on their QTL-allele constitutions (Figure 2E with the allele effect expressed in dark color).

The varieties with low DLP had more alleles with negative effects than those with high DLP (Figure 2E). The phenomenon was also observed from the QTL-allele matrix of 5 highly susceptible and 10 highly resistant accessions (Figure 2F). It can be found that the two groups shared the same alleles on 18 loci (3 alleles with positive effects and 15 alleles with negative effects), and differentiation existed on 48 loci (Table S4). The average number of alleles with negative effects in the highly resistant accession group was 39.3 per accession, ranging from 37 to 43, while in the highly susceptible accession group, it was 33.6 per accession, ranging from 31 to 37. The average of the allele effect value of 10 highly resistant accessions (low DLP) on 66 main-effect QTLs was −34.3 per accession, ranging from −25.1 to −42.0, while that of the 5 highly susceptible accessions was 6.2 per accession, ranging from −4.4 to 11.2. The landrace S02 with the lowest sum of allele effects of −42.0 was composed of 42 negative alleles and 24 positive alleles, while the landrace S11 with the highest sum of allele effects of 11.2 was composed of 33 negative alleles and 33 positive alleles (Table S4). This indicated that the accumulation of alleles with negative effects has potential regarding improvements in the antixenosis of the CSLRP.

### 2.3. Prediction of Recombination Potential and Optimal Crosses for Antixenosis in CSLRP

Using the QTL-allele matrix to predict the recombination potential and optimal crosses in CSLRP, all possible crosses (68,265), each comprising 2000 homozygous progenies, were simulated under a linkage and independent assortment model, respectively (Figure 2G). The 25th percentile of the cross progeny was used as an indicator to measure the recombination potential among alleles for antixenosis in CSLRP (Figure 2G). This indicator is a moderate requirement for the CSLRP because the inconvenience of comparisons among negative recombination values caused by high selection pressure is to be avoided. The 25th percentile DLP of the simulated 68,265 crosses under the linkage model ranged from 3.0% to 71.7%, with an average of 37.0%, and 875 crosses were lower than the minimum DLP (17.2%) in CSLRP (Table 3). Therefore, the recombination potentials of the 25th percentile DLP in all possible crosses were predicted as 44.5% − 37.0% = 7.5% on average, 44.5% − 3.0% = 41.5% as the maximum improvement and 17.2% − 3.0% = 14.2% as the maximum transgressive potential (Table 1 and Table 3). For the ecoregions, 9, 79, 140, 106, 23 and 70, predicted crosses showed lower than the minimum DLP within ER-I to ER-VI in the CSLRP. The predicted minimum DLP of all crosses varied greatly within the ecoregions, ranging from 5.1% (ER-IV) to 20.4% (ER-V), much lower than the minimum accession (17.2%) in the CSLRP. There were 180 crosses within ecoregions showing a lower DLP than the minimum accession, while 695 crosses among ecoregions showed a lower DLP than the minimum accession, indicating great transgressive potential within and among ecoregions (Table 3).

Table 4 shows the DLP values of the predicted top 20 crosses in the CSLRP, which were recommended for CCW-antixenosis breeding programs. These crosses showed that all the parents were RVs (DLP 17.2~29.4%) but not necessarily the best ones; there were significant transgressive segregations under less serious selection pressure at the 25th percentile. More recombination potential can be expected under a slight increase in selection pressure at the 20th percentile. Therefore, quite large recombination potential exists for an improvement in antixenosis to CCW in the CSLRP.

In addition, the predicted recombination potentials under the linkage model were not much different from those under the independent assortment model, which indicates there is no further linkage barrier to be broken for releasing further recombination potential in the genetic system of antixenosis against CCW in the CSLRP.

### 2.4. Candidate Gene Annotation of Antixenosis in CSLRP

Around the 86 identified DLP QTLs for antixenosis, there were 769 possible genes located, in which 607 genes had no SNP significantly linked to the associated SNPLDBs, and the other 162 genes contained 300 SNPs significantly linked to identified SNPLDBs according to SoyBase (http://www.soybase.org (accessed on 1 September 2022)). Finally, a total of 62 candidate genes were inferred, explaining 31.29% phenotypic variation. Therefore, a series of genes, rather than a few, conferred antixenosis against CCW. The Gene Ontology (GO) enrichment analysis revealed that the 62 genes were involved in eight categories of biological processes (Figure 2H, Table 5).

Category I contained 13 genes (20.97% of the 62 genes, accounting for 9.04% PV) associated with the response to abiotic and biotic stresses. Plants have various response mechanisms that allow them to cope with abiotic and biotic stresses.

Category II contained 14 genes (25.58%, accounting for 5.56% PV) associated with secondary metabolism, including the hydrogen peroxide biosynthetic process, polyamine biosynthetic process, indoleacetic acid biosynthetic process, jasmonic acid biosynthetic process, oxidation reduction process, flavonoid biosynthetic process, selenium compound metabolic process, toxin catabolic process and xylan biosynthetic process. Some secondary metabolite biosynthesis pathways contain insect-resistance-related genes, such as the hydrogen peroxide biosynthetic process, flavonoid biosynthetic process and jasmonic acid biosynthetic process [37,38,39].

Category III contained six genes (9.68%, accounting for 4.67% PV) associated with primary metabolism, which can provide the necessary metabolites and energy for life activities.

Category IV contained five genes (8.06%, accounting for 2.69% PV) associated with signal transduction, which may play an important role in CCW resistance.

Category V contained four genes associated with transport, which contribute to the maintenance of normal life activities and ensure normal material exchange, information transmission and other functions.

Category VI contained five genes associated with cell growth, which help to promote organ differentiation related to antixenosis.

Category VII contained three genes associated with DNA metabolism, which contribute to the regulation of biological genetic information and play an important role in gene expression regulation and developmental regulation.

Category VIII contained 12 genes associated with unknown processes.

A trait of antixenosis against CCW conferred by 62 genes involving eight functional categories, in fact, composes a gene network, which is to be further studied for interrelationships. Among the candidate genes, *Glyma08g19650* in *q-DLP-08-2*, *Glyma08g43830* in *q-DLP-08-7*, *Glyma10g19710* in *q-DLP-10-4*, *Glyma13g10070* in *q-DLP-13-3*, *Glyma13g26030* in *q-DLP-13-8*, *Glyma15g37276* in *q-DLP-15-2*, *Glyma17g16641* in *q-DLP-17-1* and *Glyma18g46220* in *q-DLP-18-4* are genes located in LC QTLs (*R*^2^ ≥ 1%), involved in biological processes IV, V, VIII, II, VIII, I, VII, I and I, respectively. These eight genes may be important candidate genes for antixenosis to CCW in CSLRP (Table 5); three of them are involved in the response to biotic and abiotic stresses (category I).

### 2.5. Genetic Structure Differentiation among Antixenosis Degree Groups in CSLRP

The accessions in the CSLRP were classified into three groups of antixenosis degrees, i.e., susceptible varieties (SV, bottom 20% accessions with DLP of 55.17–79.88%), moderately resistant varieties (MRV, middle 60% accessions with DLP of 33.81–55.16%) and resistant varieties (RV, top 20% accessions with DLP of 17.23–33.73%). The QTL-allele matrix can be separated into SV, MRV and RV sub-matrices, from which the genetic changes in antixenosis from SV to MRV and then to RV can be traced to understand the genetic constitutions of different degree categories of antixenosis (Table 6 and Table S5).

The statistics of the changed alleles show that QTL-alleles changed from SV (186 alleles with 93 negative and 93 positive ones) to MRV (202 alleles with 104 negative and 98 positive ones) and then to RV (187 with 102 negative and 85 positive ones) on 66 loci (Table 6 and Table S5). In MRV and RV, 185 and 172 alleles were the same as in SV; only 18 and 29 alleles were different, in which 17 and 15 alleles were increased, while 1 and 14 alleles were decreased, respectively. In comparison, between RV and MRV, 186 alleles (102 negative and 84 positive ones) of the former were the same as in the latter on 66 loci, 1 increased allele on 1 locus and 16 decreased alleles on 15 loci in RV (Table 6 and Table S5). The QTL-allele structure changes from SV to MRV and then to RV indicate that, along with the soybean varieties’ antixenosis being enhanced, the allele constitution was also changed, with more negative alleles increasing and positive alleles decreasing.

All increased or decreased alleles and their corresponding QTLs are listed in Table 7. For the main-effect QTL-alleles in MRV and RV compared to SV, 17 alleles increased on 14 loci, while 15 alleles decreased on 14 loci. Among the increased alleles, 11 had negative effects, especially those of the three LC-QTLs (*R*^2^ ≥ 1%) *q-DLP-08-7*, *q-DLP-10-4* and *q-DLP-13-3,* with large negative allele effects, −11.02, −6.51 and −6.53, respectively, while among the decreased alleles, 14 had positive effects. These alleles should benefit the antixenosis to CCW in CSLRP. In addition, in the QTLs *q-DLP-06-6* and *q-DLP-13-3*, each had three changed alleles (negative and positive); both had increased and decreased alleles from SV to MRV and RV, indicating that the allele change was not necessarily only in a single direction on the same locus. The frequency of the increased and decreased alleles on a locus in MRV + RV ranged between 1.35% and 7.77% and between 0.34% and 2.70%, respectively, which is not very large, indicating no serious selection pressure was experienced in the antixenosis enhancement (Table 7).

Integrating the QTL-alleles with the annotated genes, which changed from susceptible to moderate and resistant varieties in CSLRP, 26 QTLs with 32 QTL-alleles were involved, in which 19 genes were annotated, involving 25 QTL-alleles (Table 7). Here, the gene alleles were not identified for individual genes due to the genes being annotated from QTLs that were not from direct mapping procedures. These changed genes and QTL-alleles included three genes (three QTL-alleles) in Category I (response to stress), six genes (nine QTL-alleles) in Category II (secondary metabolism), four genes (six QTL-alleles) in Category III (primary metabolism), one gene (one QTL-allele) in Category IV (signal transduction), two genes (two QTL-alleles) in Category V (transport) and three genes (four QTL-alleles) in Category VIII (unknown process), not including Category VI (cell growth) and Category VII (DNA metabolism). Among the 25 QTL-alleles, 8 had increased negative alleles on 7 loci, and 11 had decreased positive alleles on 11 loci (Table 5 and Table 7). These are, in fact, the major genetic constitution differences for the antixenosis variation between SV and MRV + RV at the seedling stage or, in other words, the genetic mechanism of the antixenosis enhancement in the CSLRP.

## 3. Discussion

### 3.1. Efficiency of the Seedling Stage Mini-Tray Identification System and Relative Consistency of Antixenosis between the Seedling and Adult Stage

Xing et al. [2] reported a seedling stage mini-tray identification system, which tested materials in seedling trays in a small net room in a greenhouse. This method not only reduced the workload and cost and shortened the cycle of insect resistance identification but also effectively solved the problem of the large demand for CCW larvae. However, it remains to be proved whether the antixenosis at the seedling stage in the mini-tray is consistent with the antixenosis at the adult stage in a field plot. In the present study, the seedling stage mini-tray identification technique was used and compared to the normal procedure at the adult stage in the field. The correlation of DLP between antixenosis at the seedling and adult stages was 0.82 **, while the correlations of antibiosis between larva weight at the seedling stage and consumed leaf amount, body weight increase and excrement amount were 0.85 **, 0.71 ** and 0.69 **, respectively, indicating that the antixenosis performance at the seedling and adult stages was relatively consistent (Table S3), as was the case for antibiosis. Thus, the antixenosis evaluation at the seedling stage may replace that at the adult stage to reduce the workload and costs. As for antibiosis, it must be studied further, since antibiosis is a complex trait.

Antixenosis, antibiosis and tolerance are three different resistance mechanisms. A variety may have more than one resistance mechanism or even different mechanism combinations [34,40,41,42,43]. In this case, the varieties often show broad-spectrum and durable resistance [44]. Particularly, under natural field conditions, varieties with only a single resistance mechanism might lose the resistance as time goes on due to the pest adaptation process. Therefore, breeders are pursuing complex insect resistance mechanisms.

### 3.2. Important QTLs-Alleles and Candidate Genes of Antixenosis against CCW in CSLRP

Previous studies mainly focused on finding a few insect resistance genes, while this study focused on a gene system in a germplasm population [45,46,47,48]. In the above text, 82 QTLs (62 genes) were identified, in which the 8 LC-QTLs with their corresponding eight genes were nominated as important QTLs/genes involved in six GO categories, with Category I seen most often. Moreover, in the genetic structure changes from SV to MRV and RV—32 alleles on 26 QTLs related to 19 annotated genes—a series of QTL-alleles/genes were involved in the antixenosis enhancement, which is important to note. These genes and QTL-alleles changed due to an antixenosis improvement involving six of eight functional categories, excepting Category VI (cell growth) and Category VII (DNA metabolism); response to stress, primary metabolism and secondary metabolism occurred more often. Among the changed QTLs/genes, five, i.e., *q-DLP-08-7* (*Glyma08g43830*, transport function), *q-DLP-10-4* (*Glyma10g19710*, unknown process function), *q-DLP-13-3* (*Glyma13g10070*, secondary metabolism function), *q-DLP-14-1* (*Glyma14g13792*, primary metabolism function) and *q-DLP-18-4* (*Glyma18g46220*, response to stress function), were also large-contribution QTLs/genes, which should be more important than the others in terms of improvements in antixenosis (Table 7).

Furthermore, in the present study, the ten best varieties and five most susceptible ones for antixenosis were identified, with their QTL-allele constitutions revealed (Table S4). In the QTL-allele structure of the 66 main-effect loci, among the 26 changed loci, 15 (marked yellow) showed variation among the selected 10 resistant and 5 susceptible varieties, and 11 loci (marked green) remained without variation, while the other 40 loci (without color) were not related to antixenosis changes; some fluctuations even existed (Table S4). For the two sets of selected extreme varieties, only 15 of the 26 QTLs were important, because there was no allele variation in the 11 other loci among the 15 extreme varieties. The 15 especially important loci with 12 candidate genes, included in the QTLs in Table 7 and Table S4, therefore can be studied with priority to understand the antixenosis mechanism.

In summary of the above three criteria analyses, out of the 8, 5 and 15 QTLs (8, 5 and 12 genes), 4 QTLs/genes, *q-DLP-08-7* (*Glyma08g43830*), *q-DLP-10-4* (*Glyma10g19710*), *q-DLP-13-3* (*Glyma13g10070*) and *q-DLP-18-4* (*Glyma18g46220*), were commonly identified and, therefore, should be the most important ones in antixenosis to CCW in the CSLRP. In the literature, *Glyma08g43830* belongs to the ABC transporter protein. The intake of the ABC transporter protein may affect the expression levels of certain ABC family genes related to the resistance of pests in the midgut of larvae [49]. *Glyma13g10070* was associated with the flavonoid biosynthetic process. UDP-glycosyltransferase (UGT) is involved in imparting resistance to leaf-chewing insects by altering the flavonoid content and expression patterns of genes related to flavonoid biosynthesis and defense [38]. *Glyma18g46220* participates in cold stress, which can cause changes in various biochemical indicators, such as polyphenol oxidase (PPO) and lipoxygenase (LOX). PPO and LOX were reported to play a key role in defense responses against insects [50,51]. Regarding another of the most important genes, *Glyma10g19710*, we did not find relevant information related to insect resistance.

Altogether, the present QTLs-alleles (genes-alleles) in the CSLRP indicate different functional QTLs/genes conferring the same trait of antixenosis against CCW. This QTL/gene system and its interrelationships must be further studied. To do so, the nominated most important four genes might be a good starting point for studying the antixenosis gene system or the gene network.

In addition, compared to the literature, the QTLs for the antibiosis of CCW were reported as *CCW-1* and *CCW-2* on chromosome 7 [8], and the QTLs for antixenosis of CCW were *qrslx1* and *qrslx2* located on chromosome 7 and chromosome 12 [23] and *qrslx3* and *qrslx4* on chromosome 7 and 2 [24]. Oki et al. [52] revealed an antixenosis resistance QTL to CCW on chromosome 7, a position that was almost the same as that of *CCW-2*. But these QTLs are not included in our QTL list (Table 2), except that *q-DLP-12-8* might be close to the antixenosis QTL *qrslx2* [23]. Among the 86 QTLs as well as the 62 candidate genes, many new ones were identified in our study, since the Chinese soybean landrace population was composed of 370 varieties, covering a wide range of areas, while those reported in the above literature were usually from limited materials, such as bi-parental crosses [8,23,24,52]. In other words, the antixenosis QTLs with their alleles not included in the CSLRP might not be broadly distributed but rather only in some specific materials.

### 3.3. Promising Breeding Potential and the Novel Antixenosis Sources in CSLRP

As indicated in Section 2, from RTM-GWAS, the QTL-allele constitutions of each variety and, therefore, of the population can be expressed in a QTL-allele matrix, from which all possible crosses may be predicted. As indicated by the present results, the recombination potentials expressed in the 25th percentile DLP of all possible crosses were predicted as a reduction of 41.5% as the maximum improvement and of 14.2% as the maximum transgression. In this way, the optimal crosses were predicted based on the QTL-allele matrix, which should be efficient and effective in exploring all complementary combinations among all the DLP/antixenosis loci. In addition, the predicted best crosses and progenies were inferred from the phenotypic and genotypic evaluations, which will be demonstrated in field experiments.

Another way to design optimal crosses based on the RTM-GWAS results is to find the counterpart for an elite variety according to their QTL-allele constituents. For example, among the 10 best antixenosis varieties, 5 (i.e., S02, S05, S06, S07, and S08) with antixenosis and antibiosis at the seedling and adult stages better than Lamar (an internationally recognized resistance source for leaf-feeding insects) were selected and recommended for breeding on leaf-feeding insect resistance. To find their complementally elite alleles for further improvements in antixenosis, the QTL-allele information of the varieties in the CSLRP may be used but not necessarily limited in the CSLRP because our recommended 20 best crosses in the population did not include all 10 elite varieties as parents. That means that some of the elite varieties could not meet all their required counterpart alleles in the CSLRP. This is especially true if multiple traits are involved in a breeding program, and a counterpart could not provide elite complementary alleles for all the traits. Thus, for a breeding program, the breeders need to enrich their germplasm collection and explore novel genes–alleles continuously.

## 4. Materials and Methods

### 4.1. Source of CCW Used

The initial source of CCW larvae used in the experiment was provided by the Entomology Laboratory of Nanjing Agriculture University. They were artificially cultured and propagated to obtain sufficient third-instar larvae. The CCW population was reared in the standard insectarium, with an area of about 5 m^2^, at 28 ± 1 °C and 60–70% relative humidity, under a 16 h light/8 h dark photoperiod rhythm [6]. The standard insectarium was disinfected once every generation.

### 4.2. Plant Materials and Evaluation of Antixenosis to CCW at the Seedling Stage

The representative sample of CSLRP, composed of 370 accessions, from six ecoregions (43, 103, 71, 80, 34 and 39 from ecoregions I–VI, respectively) obtained from the National Center for Soybean Improvement of China were used in the study at Dangtu Experimental Station, Anhui, China (Table 1). The materials were tested in a mini-tray identification system proposed by Xing et al. [2], where they were sown in a 32-hole seed nursery tray (54, 28 and 6 cm in length, width and height, respectively) in an insecticide-free micro-net-room in a greenhouse in June 2017, April 2018 and September 2019. Six seeds were sown in each hole of the 32-hole tray using a randomized complete blocks design with three repetitions. No pesticide or herbicide was applied during soybean growth in the greenhouse. At the VC stage, two seedlings were kept in each hole. The artificial infestations were initiated when the first trifoliate leaves of seedlings were fully unrolled. Two third-instar CCW larvae with visually uniform size were applied to leaves of each accession in each hole. The DLP was evaluated as the indicator of antixenosis, with the leaves not fed by CCW recorded as 0%, whereas the leaves completely fed by CCW were recorded as 100%. The DLP ranged from 0 to 100%. Each replication was recorded at the early, middle and late stages, while the whole experiment average DLP reached about <30%, 40–60% and >70%, respectively.

### 4.3. Evaluation of Antixenosis and Antibiosis at the Adult Stage of Selected Materials

Ten highly resistant varieties and five highly susceptible varieties, i.e., S01–S15 (Table S2), were screened out from the CSLRP according to the antixenosis against CCW at the seedling stage. The S01–S15, with Lamar [53] as the resistant control and NN89-29 [2] as the susceptible control, were planted in hill plot in the field at a spacing of 60 cm × 20 cm, with ten seeds per hill in a net room in 2019 at Dangtu Experimental Station of Nanjing Agricultural University. No pesticide or herbicide was applied during soybean growth in the net room. At the VC stage, each plot was thinned to five plants; then, at the R1 stage, the plants were artificially infested with fifty visually uniform third-instar CCW larvae per plot using a small paint brush. The DLP was used as the indicator of antixenosis.

The plant materials for antibiosis evaluation were the same as those for the antixenosis test at the adult stage. The identification method of antibiosis at the seedling and adult stages referred to Wang et al. [54] and Hu et al. [55], respectively. The expanded trifoliate leaves on the third preapical nodes at the V5 stage were used for antibiosis evaluation at the seeding stage, while those leaves at the R2 stage were used for antibiosis evaluation at the adult stage [56]. The CCW larva weight on the 6th, 9th and 12th day was used as the indicator of antibiosis at the seeding stage, while the consumed leaf amount, body weight increase and excrement amount were used as the indicators of antibiosis at the adult stage.

### 4.4. Statistical Analysis of Phenotypic Data

The statistics of the DLP data, including frequency distribution, mean, minimum, maximum, *F*-test and coefficient of variation (*CV*), were obtained using the PROC GLM procedure in the SAS program (SAS Institute Inc., Cary, NC, USA). Duncan’s new multiple range test was used for multiple comparisons. The heritability in a single year and three years was estimated as *h*^2^ = *σ*^2^_g_/(*σ*^2^_g_ + *σ*^2^_e_/*r*) and *h*^2^ = *σ*^2^_g_/(*σ*^2^_g_ + *σ*^2^*_g_*_e_/*n* + *σ*^2^_e_/*nr*), where *h*^2^ = heritability, *σ*^2^_g_ = genotypic variance, *σ*^2^_e_ = error variance, *σ*^2^*_g_*_e_/*n* = *G × E* variance, *n* = number of environments and *r* = number of replications [57]. The genotypic coefficient of variation (*GCV*) was calculated as *GCV* = *σ*_g_/*μ*, where *μ* is the population mean. All the parameters were estimated from the expected mean squares in GLM.

The plot DLP value is a weighted average of damaged leaf percentage over three observation times. The calculation of a plot DLP involves the following: (i) calculating the coefficient of variation (*CV_i_*) of each observed value (*Y_i_*) for each of the three observation times, where *i* represents the *i*th observation time; (ii) calculating the weight (*P_i_* = *CV_i_*/Σ*CV_i_*) for each observed value at three observation times; (iii) calculating the weighted average (*PM_i_* = Σ(*P_i_* × *Y_i_*)) for each plot.

### 4.5. Genome-Wide Association Analysis Using RTM-GWAS

The genotyping method for the present population was reported previously by Meng et al. [34]. Restriction site-associated DNA sequencing (RAD-seq) was used for SNP genotyping. RealSFS software (http://popgen.dk/angsd/index.php/RealSFS (accessed on 1 September 2022)) was used for detecting SNPs; then, the detected SNPs were filtered according to the standard of missing and heterozygosity ≤ 20% and minimum allele frequency (MAF) ≥ 1% [58]. The fast PHASE software (http://scheet.org/software.html (accessed on 1 September 2022)) [59] was used for genotyping SNP imputation after heterozygous alleles were removed as missing alleles. A total of 116,769 SNPs distributed throughout the genome were obtained after quality control, and 29,234 SNPLDB markers with their 71,903 haplotypes were constructed under linkage disequilibrium *D*′ > 0.7 criterion according to the RTM-GWAS (https://github.com/njau-sri/rtm-gwas (accessed on 1 September 2022)) [30].

The association analysis was performed with a two-stage strategy: markers were first selected through a general linear model (GLM) procedure, and then the selected markers were examined further with stepwise regression under a multi-locus linear model. The markers significantly associated with antixenosis to CCW were obtained on the basis of the significant association threshold (*p* = 0.05). In the association analysis, the genetic similarity coefficient matrix based on SNPLDB markers was used to correct the population structure.

### 4.6. Optimal Cross Prediction

All possible single crosses among the 370 accessions were generated in silico under a linkage model and independent assortment model for the recombination potential of antixenosis [30]. For each cross, the predicted genotypic antixenosis value was calculated from 2000 continuously inbred progenies derived from F_2_ individuals based on the QTL-allele matrix. The 25th percentile of a cross was used as its predicted cross value.

### 4.7. Candidate Gene Function Annotation

The candidate genes were annotated from the detected QTL system in the following way: (1) identifying genes within 50 kb, flanking the associated SNPLDBs according to the information provided in SoyBase (http://www.soybase.org (accessed on 1 September 2022)); (2) selecting the candidate gene among the above-identified genes using the Chi-square test for the most significant association between the detected SNPLDB and SNPs in a gene at the *p* = 0.05 significance level. The annotations and calls of candidate genes were retrieved from the Wm82.a1.v1.1 gene model in SoyBase (http://www.soybase.org (accessed on 1 September 2022)).

## Figures and Tables

**Table 1:** Frequency distribution and descriptive statistics of damaged leaf percentage at the seedling stage in CSLRP (%).

Eco-Region	Class Midpoint of DLP														N	Mean	Min.	Max.	*F*	*h* ^2^	*GCV*	*CV*
	15	20	25	30	35	40	45	50	55	60	65	70	75	80								
All	1	6	28	30	45	62	47	50	44	27	18	7	4	1	370	44.5	17.2	79.9	6.2 **	77.3	24.1	19.1
ER-I		1	2	5	4	7	6	6	7	3			2		43	44.6 ab	18.9	74.9	3.7 **	63.3	21.7	24.7
ER-II	1	1	11	11	14	18	12	12	14	7		2			103	42.0 b	17.2	69.5	5.3 **	72.1	22.9	20.3
ER-III				3	8	16	8	8	10	6	10	2			71	49.3 a	30.2	70.3	5.9 **	75.9	19.7	16.2
ER-IV		2	6	4	9	9	10	13	10	5	7	2	2	1	80	46.9 a	19.8	79.9	9.3 **	83.3	13.6	16.3
ER-V			3	2	6	8	4	8		3					34	41.7 b	25	62.1	5.7 **	77.5	20.1	16.6
ER-VI		2	6	5	4	4	7	3	3	3	1	1			39	40.2 b	20.3	67.5	6.6 **	80.1	28.4	21.4

Note: DLP is damaged leaf percentage; plot DLP is the mean of the weighted average of DLP, which was measured at seedling stage in 2017, 2018 and 2019. Mean, Min, Max, *F*, *h*^2^, *GCV* and *CV* represent the mean, minimum, maximum, *F* value, heritability, genetic coefficient of variation and error coefficient of variation, respectively. ** Indicates significance at 0.01 probability level. ER: ecoregion; ER-I: Northern Single Cropping Spring Planting Varietal Ecoregion; ER-II: Huang-Huai-Hai Double-Cropping Spring and Summer Planting Varietal Ecoregion; ER-III: Middle and Lower Changjiang Valley Double-Cropping Spring and Summer Planting Varietal Ecoregion; ER-IV: Central South Multiple-Cropping Spring Summer and Autumn Planting Varietal Ecoregion; ER-V: Southwest Plateau Double-Cropping Spring and Summer Planting Varietal Ecoregion; ER-VI: South China Tropical Multiple-Cropping All-Season Planting Varietal Ecoregion. The abbreviation of ecoregion applies to the following text. Values in “Mean” column followed by different lowercase letters represent significant difference among ecoregions at the *p* ≤ 0.05 level.

**Table 2:** Identified QTLs of damaged leaf percentage at seedling stage in CSLRP.

QTL	AN	QTL Main		QTL × Env		Reported QTL/Marker
		−log_10_*p*	*R*^2^ (%)	−log_10_*p*	*R*^2^ (%)	
*q-DLP-01-1*	2	1.66	0.08			
*q-DLP-01-2*	2	1.55	0.07	4.24	0.28	*Gm01sv037* [6]
*q-DLP-01-3*	2	2.84	0.15			*Satt436* [6]
*q-DLP-01-4*	5	4.71	0.39			
*q-DLP-01-5*	2			1.42	0.09	
*q-DLP-01-6*	4	8.56	0.62	4.84	0.46	
*q-DLP-01-7*	2	3.54	0.19			Satt147 [6]/*qRWF-1* [33]
*q-DLP-02-1*	6	1.41	0.17	4.83	0.58	
*q-DLP-02-2*	4	1.67	0.14	2.77	0.30	
*q-DLP-02-3*	2	9.45	0.57			
*q-DLP-02-4*	2	2.78	0.14	2.17	0.14	
*q-DLP-02-5*	5	9.32	0.71	1.85	0.27	
*q-DLP-02-6*	2			3.57	0.24	*Satt005* [6]/*SIR-D1b* [34]
*q-DLP-02-7*	2	2.92	0.15	4.10	0.27	*Sat_289* [6]
*q-DLP-03-1*	3	4.10	0.27	1.79	0.17	
*q-DLP-03-2*	2	2.87	0.15			*Gm03sv093* [6]
*q-DLP-04-1*	2	1.97	0.09	1.81	0.12	*BP4-1* [35]
*q-DLP-04-2*	2			2.44	0.16	
*q-DLP-05-1*	2			2.29	0.15	
*q-DLP-05-2*	2			3.73	0.25	
*q-DLP-06-1*	3	7.14	0.47			*Satt681* [6]
*q-DLP-06-2*	2	1.59	0.07	2.03	0.13	*Satt489* [6]
*q-DLP-06-3*	2	4.66	0.26	3.82	0.25	*Satt489* [6]
*q-DLP-06-4*	2			1.87	0.12	
*q-DLP-06-5*	2	5.01	0.28	4.74	0.31	
*q-DLP-06-6*	7	8.74	0.75	2.67	0.44	*Sat_312* [6]
*q-DLP-06-7*	2	9.00	0.54	3.28	0.22	*BP6-2* [35]
*q-DLP-06-8*	2			2.27	0.15	
*q-DLP-07-1*	2	2.92	0.15			
*q-DLP-07-2*	2			2.67	0.18	
*q-DLP-07-3*	2			3.78	0.25	
*q-DLP-08-1*	3	2.87	0.19	2.59	0.23	
***q-DLP-08-2***	**6**	**29.24**	**2.16**	**3.60**	**0.48**	
*q-DLP-08-3*	5	11.46	0.86	2.68	0.35	
*q-DLP-08-4*	2	1.33	0.06			
*q-DLP-08-5*	2			1.86	0.12	
*q-DLP-08-6*	2	11.21	0.68			
***q-DLP-08-7***	**2**	**24.2**	**1.55**			
*q-DLP-08-8*	5	8.59	0.66	4.50	0.50	
*q-DLP-10-1*	4	10.64	0.76	2.8	0.31	
*q-DLP-10-2*	2			5.01	0.33	
*q-DLP-10-3*	2	14.02	0.87			
***q-DLP-10-4***	**6**	**19.29**	**1.46**	**2.90**	**0.42**	
*q-DLP-10-5*	2	3.60	0.19			*BP10-2* [35]
*q-DLP-10-6*	2			2.03	0.13	
*q-DLP-10-7*	2			6.21	0.41	
*q-DLP-11-1*	2			3.01	0.20	
*q-DLP-11-2*	5	2.56	0.23			
*q-DLP-11-3*	2			2.04	0.13	
*q-DLP-12-1*	2	5.98	0.34			
*q-DLP-12-2*	2	2.71	0.14			
*q-DLP-12-3*	2	1.87	0.09			
***q-DLP-12-4***	**5**	**71.48**	**5.16**	**2.43**	**0.33**	
*q-DLP-12-5*	2			2.38	0.16	
*q-DLP-12-6*	2	6.53	0.38			
*q-DLP-12-7*	2	5.37	0.30	2.71	0.18	
*q-DLP-12-8*	2	2.48	0.12	3.44	0.23	
*q-DLP-13-1*	2	13.42	0.83			
*q-DLP-13-2*	2	2.69	0.14			
***q-DLP-13-3***	**11**	**22.1**	**1.88**	**7.25**	**1.06**	
*q-DLP-13-4*	2	6.05	0.35	2.84	0.19	
*q-DLP-13-5*	2			3.10	0.20	
*q-DLP-13-6*	2	5.67	0.32			
*q-DLP-13-7*	2			7.12	0.47	
***q-DLP-13-8***	**3**	**14.96**	**1.00**	**2.52**	**0.23**	*QTL_13_1* [36]
***q-DLP-14-1***	**7**	**17.82**	**1.40**	**2.95**	**0.47**	
*q-DLP-14-2*	2	1.98	0.09			
*q-DLP-14-3*	2	3.25	0.17			*Satt020* [6]
***q-DLP-15-1***	**2**	**35.9**	**2.35**	**1.86**	**0.12**	
***q-DLP-15-2***	**4**	**43.98**	**3.08**	**8.68**	**0.75**	*Gm15sv557* [6]
*q-DLP-15-3*	2	3.87	0.21	5.13	0.34	
*q-DLP-15-4*	2			1.98	0.13	
***q-DLP-17-1***	**4**	**27.4**	**1.91**	**4.60**	**0.45**	
*q-DLP-17-2*	2	1.33	0.06	2.27	0.15	
*q-DLP-18-1*	2			2.44	0.16	
*q-DLP-18-2*	2	2.06	0.10	3.79	0.25	*Gm18sv664* [6]
*q-DLP-18-3*	2	6.62	0.38			*Gm18sv704* [6]
***q-DLP-18-4***	**7**	**32.42**	**2.44**			*Gm18sv704* [6]
*q-DLP-18-5*	2	3.75	0.20	1.31	0.09	*Gm18sv704* [6]
*q-DLP-18-6*	2	2.81	0.14			
*q-DLP-18-7*	2	1.67	0.08	3.14	0.21	
***q-DLP-19-1***	**4**	**15.13**	**1.07**	**3.76**	**0.38**	
*q-DLP-19-2*	2	2.46	0.12	2.35	0.16	
*q-DLP-19-3*	2	2.33	0.11			
*q-DLP-20-1*	2	2.02	0.10			
*q-DLP-20-2*	3	13.17	0.88			*GMES0205* [6]
LC-QTL		12 (61) ^a^	25.45	1 (6)	1.06	
SC-QTL		54 (142)	16.55	56 (166)	14.79	
Total 86	243	66 (203)	42.00	57 (172)	15.85	

Note: QTL: QTL name, such as *q-DLP-12-4*, is designated in the following way: *q* means QTL; DLP represents damaged leaf percentage, 12 means chromosome 12, and 4 means its order on the chromosome according to its physical position. QTL in boldface means the locus is a large-contribution QTL (LC-QTL) with an *R*^2^ (contribution to phenotypic variance) of more than 1%, while a QTL in normal font means a small-contribution QTL (SC-QTL). AN: allele number in a QTL in CSLRP. QTL main: main-effect QTL; QTL × Env: a QTL interacting with the environment (QEI). *R*^2^: phenotypic contribution of a QTL. ^a^ 12 (61): the number outside of the parentheses is the number of QTLs, while that in parentheses is its total alleles.

**Table 3:** The predicted 25th percentile DLP of single crosses under the linkage and independent assortment model in ecoregions and the CSLRP.

ER	Minimum	Total No. of Crosses	Predicted DLP (%)			No. of Optimal Crosses
	DLP (%)		Mean	Min.	Max.	
ER-I	18.9	903	36.6 (36.3)	12.6 (10.4)	62.3 (60.6)	9 (16)
ER-II	17.2	5253	34.3 (33.9)	9.4 (10.3)	62.4 (60.3)	79 (85)
ER-III	30.2	2485	42.1 (41.4)	20.2 (16.8)	65.5 (65.5)	140 (191)
ER-IV	19.8	3160	39.1 (39.1)	5.1 (8.9)	71.1 (71.3)	106 (83)
ER-V	25.0	561	35.3 (35.1)	20.4 (20.5)	55.8 (56.5)	23 (30)
ER-VI	20.3	741	33.0 (32.4)	5.7 (9.5)	59.1 (59.9)	70 (73)
Within	17.2	13,103	37.1 (36.7)	5.1 (8.9)	71.1 (71.3)	180 (171)
Between	17.2	55,162	37.0 (36.6)	3.0 (6.7)	71.7 (70.6)	695 (659)
All	17.2	68,265	37.0 (36.6)	3.0 (6.7)	71.7 (71.3)	875 (830)

Note: ER: ecoregion; see the note in Table 1 for ecoregion names. Minimum DLP: the minimum of observed DLP in the corresponding ecoregion. Predicted DLP is the 25th percentile of predicted DLP in a simple cross. No. of optimal crosses means the number of crosses with the 25th percentile DLP value lower than the minimum DLP in the ecoregion. “Within” means within all ecoregions. “Between” means the crosses between accessions from different ecoregions. “All” means the total crosses among all accessions in CSLRP. The numbers outside parentheses are the predicted value based on the linkage model, while those in parentheses are based on the independent assortment model.

**Table 4:** The DLP values of the predicted top 20 optimal crosses under the linkage and independent assortment model in CSLRP (%).

Code	Parent 1	Parent 2	DLP of Parent 1	DLP of Parent 2	Predicted DLP		
					Mean	25th Percentile	20th Percentile
1	S04	4L367	17.2	21.1	18.5 (19.3)	3.0 (6.7)	0.1 (3.9)
2	S03	4L367	19.8	21.1	20.4 (20.0)	3.7 (7.5)	0.2 (4.3)
3	S03	S06	19.8	23.1	20.5 (20.6)	5.1 (8.9)	1.3 (6.1)
4	S04	S06	17.2	23.1	19.8 (19.6)	5.6 (7.0)	2.4 (4.2)
5	4L262	4L367	20.3	21.1	21.1 (20.4)	5.7 (9.5)	3.3 (7.2)
6	4L250	4L367	24.1	21.1	23.0 (22.1)	5.9 (10.3)	3.0 (7.5)
7	4L187	4L367	25.6	21.1	22.9 (24.1)	6.2 (11.5)	3.1 (8.8)
8	4L088	4L367	25.7	21.1	23.5 (23.2)	6.3 (9.7)	2.8 (6.9)
9	S03	4L242	19.8	29.4	24.0 (24.8)	6.6 (12.1)	3.4 (8.9)
10	S04	4L242	17.2	29.4	23.5 (23.5)	7.1 (10.9)	3.7 (7.9)
11	4L074	4L367	23.3	21.1	21.8 (22.4)	7.3 (11.2)	5.6 (8.3)
12	4L263	4L367	26.1	21.1	23.3 (23.3)	7.5 (11.8)	4.7 (9.4)
13	4L261	4L367	23.6	21.1	22.0 (22.5)	7.6 (10.2)	3.8 (6.9)
14	S06	4L250	23.1	24.1	23.4 (23.2)	7.6 (11.0)	5.6 (8.7)
15	4L090	4L367	28.2	21.1	24.8 (24.3)	7.8 (12.3)	4.8 (8.9)
16	4L041	S04	27.0	17.2	21.5 (22.8)	7.9 (8.7)	4.7 (5.8)
17	S06	4L262	23.1	20.3	21.8 (22.6)	7.9 (11.6)	5.1 (9.0)
18	S05	4L367	27.2	21.1	24.7 (24.4)	7.9 (13.5)	5.9 (11.0)
19	S10	4L367	24.2	21.1	22.6 (22.9)	8.0 (11.2)	5.5 (8.2)
20	S02	4L367	18.9	21.1	19.8 (19.9)	8.0 (8.6)	5.1 (5.8)

Note: S02 = LJHD; S03 = AYLLZ; S04 = AYXHD; S05 = HZHD; S06 = STXHD; S10 = LWBMLYH; 4L041 = BYHD; 4L074 = LSHDD; 4L088 = KFGZQD; 4L090 = SJZPD; 4L187 = SJZPD; 4L242 = WXBDD; 4L250 = LJCHD; 4L261 = CMSD; 4L262 = MZXQD; 4L263 = XHHD; 4L367 = LRD-6. The numbers outside parentheses in column “Predicted DLP” are the predicted value based on the linkage model, while those in parentheses are based on the independent assortment model. The most resistant parent is that with a DLP of 17.2%; all the selected optimal crosses have their 25th and 20th percentile DLP lower than their parents (transgressive antixenosis, better than the best one in CSLRP).

**Table 5:** Sixty-two candidate genes annotated from the 86 antixenosis QTLs against CCW in CSLRP.

QTL	Position (bp)	Candidate Gene	Gene Ontology Description (Category)
*q-DLP-01-1*	2,018,307	*Glyma01g02525*	Xylan biosynthetic process (II)
*q-DLP-01-2*	45,358,479		
*q-DLP-01-3*	48,727,687	*Glyma01g36200*	Response to salicylic acid stimulus (I)
*q-DLP-01-4*	49,414,457_49,512,678	*Glyma01g37020*	Regulation of meristem growth (VI)
*q-DLP-01-5*	50,563,918_50,575,958	*Glyma01g38470*	Response to auxin stimulus (I)
*q-DLP-01-6*	51,562,541_51,627,699	*Glyma01g39590*	Xylan biosynthetic process (II)
*q-DLP-01-7*	52,647,305	*Glyma01g41110*	Hormone-mediated signaling pathway (IV)
*q-DLP-02-1*	1,973,000_2,157,204	*Glyma02g02860*	Toxin catabolic process (II)
*q-DLP-02-2*	4,018,617_4,025,103	*Glyma02g04965*	Response to chitin (I)
*q-DLP-02-3*	14,648,440_14,667,968	*Glyma02g16260*	Response to abscisic acid stimulus (I)
*q-DLP-02-4*	17,944,684		
*q-DLP-02-5*	25,406,263_25,479,114		
*q-DLP-02-6*	29,142,262_29,263,830	*Glyma02g27950*	Unknown process (VIII)
*q-DLP-02-7*	51,455,660	*Glyma02g48060*	Transmembrane transport (V)
*q-DLP-03-1*	5,122,536_5,122,820	*Glyma03g04920*	Lipid transport (V)
*q-DLP-03-2*	14,208,130		
*q-DLP-04-1*	7,402,649	*Glyma04g09220*	Unknown process (VIII)
*q-DLP-04-2*	46,268,205_46,268,222	*Glyma04g40121*	Regulation of transcription, DNA-dependent (VII)
*q-DLP-05-1*	18,014,789		
*q-DLP-05-2*	24,588,612		
*q-DLP-06-1*	420,122_487,621	*Glyma06g00730*	Response to insect (I)
*q-DLP-06-2*	22,275,863	*Glyma06g24701*	Unknown process (VIII)
*q-DLP-06-3*	24,692,151		
*q-DLP-06-4*	29,026,459		
*q-DLP-06-5*	32,468,737		
*q-DLP-06-6*	35,040,678_35,236,874	*Glyma06g33880*	Protein glycosylation (III)
*q-DLP-06-7*	44,178,948		
*q-DLP-06-8*	50,304,823_50,305,172	*Glyma06g47920*	Unknown process (VIII)
*q-DLP-07-1*	28,717,244		
*q-DLP-07-2*	36,293,327_36,297,011	*Glyma07g31320*	Protein complex assembly (III)
*q-DLP-07-3*	40,096,883	*Glyma07g35023*	Unknown process (VIII)
*q-DLP-08-1*	14,753,730_14,760,906	*Glyma08g19560*	Response to wounding (I)
***q-DLP-08-2***	**14,777,419_14,822,821**	***Glyma08g19650***	**Sugar-mediated signaling pathway (IV)**
*q-DLP-08-3*	22,259,705_22,457,223	*Glyma08g28111*	Transcription, DNA-dependent (VII)
*q-DLP-08-4*	31,137,298		
*q-DLP-08-5*	40,375,262	*Glyma08g40646*	Signal transduction (IV)
*q-DLP-08-6*	41,582,869	*Glyma08g41670*	Hydrogen peroxide biosynthetic process (II)
***q-DLP-08-7***	**43,612,649**	***Glyma08g43830***	**Transmembrane transport (V)**
*q-DLP-08-8*	45,217,877_45,266,178	*Glyma08g45950*	Response to wounding (I)
*q-DLP-10-1*	2,137,913_2,266,216	*Glyma10g03140*	Regulation of flavonoid biosynthetic process (II)
*q-DLP-10-2*	2,777,471	*Glyma10g03720*	Response to jasmonic acid stimulus (I)
*q-DLP-10-3*	4,019,592	*Glyma10g05160*	Xylan biosynthetic process (II)
***q-DLP-10-4***	**24,526,775_24,706,774**	***Glyma10g19710***	**Unknown process (VIII)**
*q-DLP-10-5*	39,555,777	*Glyma10g30930*	Unknown process (VIII)
*q-DLP-10-6*	45,227,388		
*q-DLP-10-7*	46,996,455	*Glyma10g39250*	Unknown process (VIII)
*q-DLP-11-1*	4,931,380	*Glyma11g07080*	Indoleacetic acid biosynthetic process (II)
*q-DLP-11-2*	28,787,840_28,938,957	*Glyma11g28580*	Unknown process (VIII)
*q-DLP-11-3*	35,388,102		
*q-DLP-12-1*	4,069,210_4,160,826	*Glyma12g05960*	Sugar-mediated signaling pathway (IV)
*q-DLP-12-2*	5,802,081	*Glyma12g08080*	Regulation of meristem growth (VI)
*q-DLP-12-3*	6,664,148_6,664,175	*Glyma12g08900*	Response to oxidative stress (I)
*q-DLP-12-4*	10,441,681_10,513,287		
*q-DLP-12-5*	14,076,555		
*q-DLP-12-6*	21,170,928		
*q-DLP-12-7*	28,906,122		
*q-DLP-12-8*	31,830,778	*Glyma12g28490*	Selenium compound metabolic process (II)
*q-DLP-13-1*	5,519,025		
*q-DLP-13-2*	7,661,807_7,661,825	*Glyma13g07530*	Unknown process (VIII)
***q-DLP-13-3***	**11,608,283_11,644,846**	***Glyma13g10070***	**Positive regulation of flavonoid biosynthetic process (II)**
*q-DLP-13-4*	16,616,018_16,616,049		
*q-DLP-13-5*	22,285,871		
*q-DLP-13-6*	22,414,892	*Glyma13g18720*	Plant-type cell wall modification (VI)
*q-DLP-13-7*	25,945,441	*Glyma13g22450*	Sugar-mediated signaling pathway (IV)
***q-DLP-13-8***	**29,302,932_29,302,990**	***Glyma13g26030***	**Unknown process (VIII)**
*q-DLP-14-1*	13,354,054_13,553,649	*Glyma14g13792*	Histone H3-K4 methylation (III)
*q-DLP-14-2*	15,737,492	*Glyma14g14990*	Jasmonic acid biosynthetic process (II)
*q-DLP-14-3*	43,771,273		
*q-DLP-15-1*	1,215,528	*Glyma15g01790*	Protein ubiquitination (III)
***q-DLP-15-2***	**42,911,867_42,923,525**	***Glyma15g37276***	**Defense response to fungus (I)**
*q-DLP-15-3*	47,989,791_47,999,025	*Glyma15g41020*	Regulation of meristem growth (VI)
*q-DLP-15-4*	48,785,322_48,785,485	*Glyma15g41640*	Oxidation-reduction process (II)
***q-DLP-17-1***	**13,426,556_13,441,932**	***Glyma17g16641***	**Regulation of transcription, DNA-dependent (VII)**
*q-DLP-17-2*	32,299,189	*Glyma17g29620*	Protein N-linked glycosylation (III)
*q-DLP-18-1*	3,801,888	*Glyma18g05090*	Positive regulation of cell proliferation (VI)
*q-DLP-18-2*	12,477,590		
*q-DLP-18-3*	55,745,667	*Glyma18g46050*	Defense response (I)
***q-DLP-18-4***	**55,988,107_55,988,755**	***Glyma18g46220***	**Response to cold (I)**
*q-DLP-18-5*	56,055,671	*Glyma18g46286*	Transmembrane transport (V)
*q-DLP-18-6*	58,793,224	*Glyma18g49390*	Unknown process (VIII)
*q-DLP-18-7*	60,245,212	*Glyma18g51380*	Polyamine biosynthetic process (II)
*q-DLP-19-1*	5,454,340_5,454,561		
*q-DLP-19-2*	43,592,061	*Glyma19g36280*	Xylan biosynthetic process (II)
*q-DLP-19-3*	47,642,370	*Glyma19g41400*	Protein glycosylation (III)
*q-DLP-20-1*	16,840,184	*Glyma20g11950*	Oxidation-reduction process (II)
*q-DLP-20-2*	35,128,616_35,247,501	*Glyma20g25418*	Systemic acquired resistance (I)

Note: Position: means the physical position of a locus in bp on the corresponding chromosome. The numbers in parentheses of Column “Gene ontology description” represent the biological process category of the gene conferring DLP. Here Category I is response to stress with 13 candidate genes; Category II is secondary metabolism with 14 candidate genes; Category III is primary metabolism with 6 candidate genes; Category IV is signal transduction with 5 candidate genes; Category V is transport with 4 genes; Category VI is cell growth with 5 genes; Category VII is DNA metabolism with 3 genes; Category VIII is unknown process with 12 genes. The eight QTLs in boldface are LC-QTLs.

**Table 6:** The allele changes in antixenosis from susceptible to moderate and resistant varieties in CSLRP.

Antixenosis	Total Alleles		Inherent Alleles		Changed Alleles		Allele Increased		Allele Decreased	
Degree	Allele No.	QTL No.	Allele No.	QTL No.	Allele No.	QTL No.	Allele No.	QTL No.	Allele No.	QTL No.
SV	186 (93, 93)	66								
MRV vs. SV	202 (104, 98)	66	185 (93, 92)	66	18 (11, 7)	15	17 (11, 6)	14	1 (0, 1)	1
RV vs. MRV	187 (102, 85)	66	186 (102, 84)	66	17 (2, 15)	16	1 (0, 1)	1	16 (2, 14)	15
RV vs. SV	187 (102, 85)	66	172 (92, 80)	66	29 (11, 18)	23	15 (10, 5)	12	14 (1, 13)	13

Note: The antixenosis degree of varieties was classified into three groups: susceptible varieties (SV, the top 20% DLPs), moderately resistant varieties (MRV, the middle 60% DLPs), and resistant varieties (RV, the bottom 20% DLPs). In Allele No. columns, the number outside the parentheses is the number of alleles, and the numbers inside the parentheses are the numbers of negative and positive alleles, respectively. All the allele numbers listed in each column are those of the antixenosis degree group (the former) compared to their counterparts (the latter); the allele numbers listed in the “Total Alleles” column is the former of the antixenosis degree group.

**Table 7:** Increased and decreased alleles conferring antixenosis changes from susceptible to moderate and resistant varieties.

QTL	*R*^2^ (%)	Allele	Effect	Frequency (%)					Annotated Gene (Category)
				Entire	SV	MRV	RV	MRV + RV	
*q-DLP-01-7*	0.19	a1	−7.01	1.08	0	0.90	2.70	1.35	*Glyma01g41110* (IV)
*q-DLP-02-1*	0.17	a4	−0.26	2.97	0	3.15	5.41	3.72	*Glyma02g02860* (II)
*q-DLP-02-4*	0.14	a1	−2.35	1.35	0	2.25	0	1.69	
*q-DLP-02-5*	0.71	a3	0.36	6.22	0	4.05	18.92	7.77	
*q-DLP-02-7*	0.15	a2	4.92	1.62	2.70	1.80	0	1.35	*Glyma02g48060* (V)
*q-DLP-04-1*	0.09	a2	2.65	1.35	2.70	1.35	0	1.01	*Glyma04g09220* (VIII)
*q-DLP-06-3*	0.26	a2	7.47	1.35	4.05	0.90	0	0.68	
*q-DLP-06-6*	0.75	a1	−7.95	1.35	0	1.80	1.35	1.69	*Glyma06g33880* (III)
		a2	−1.42	1.89	1.35	2.70	0	2.03	
		a7	5.88	1.89	2.70	2.25	0	1.69	
*q-DLP-08-4*	0.06	a1	−2.76	1.08	0	0.90	2.70	1.35	
***q-DLP-08-7***	1.55	a1	−11.02	2.16	0	0.90	8.11	2.70	*Glyma08g43830* (V)
*q-DLP-08-8*	0.66	a5	4.76	1.35	4.05	0.90	0	0.68	*Glyma08g45950* (I)
*q-DLP-10-1*	0.76	a1	−5.06	5.68	0	6.76	8.11	7.09	*Glyma10g03140* (II)
		a2	−1.41	2.70	0	1.80	8.11	3.38	
*q-DLP-10-3*	0.87	a2	7.38	1.35	4.05	0.90	0	0.68	*Glyma10g05160* (II)
***q-DLP-10-4***	1.46	a1	−6.51	1.89	0	1.35	5.41	2.36	*Glyma10g19710* (VIII)
		a6	5.92	1.08	0	1.35	1.35	1.35	
*q-DLP-11-2*	0.23	a5	4.46	2.43	4.05	2.70	0	2.03	*Glyma11g28580* (VIII)
*q-DLP-12-6*	0.38	a2	7.09	1.08	2.70	0.90	0	0.68	
*q-DLP-12-7*	0.30	a2	6.38	1.08	4.05	0	1.35	0.34	
*q-DLP-12-8*	0.12	a2	2.53	1.89	2.70	2.25	0	1.69	*Glyma12g28490* (II)
*q-DLP-13-1*	0.83	a1	−8.46	1.08	0	0.45	4.05	1.35	
***q-DLP-13-3***	1.88	a1	−6.53	1.62	0	1.35	4.05	2.03	*Glyma13g10070* (II)
		a10	5.06	2.70	2.70	3.60	0	2.70	
		a11	6.60	2.70	0	2.70	5.41	3.38	
***q-DLP-14-1***	1.40	a7	3.65	1.35	0	1.80	1.35	1.69	*Glyma14g13792* (III)
*q-DLP-17-2*	0.06	a2	2.42	1.62	2.70	1.80	0	1.35	*Glyma17g29620* (III)
*q-DLP-18-3*	0.38	a2	7.88	1.08	1.35	1.35	0	1.01	*Glyma18g46050* (I)
***q-DLP-18-4***	2.44	a6	5.93	1.35	0	1.35	2.70	1.69	*Glyma18g46220* (I)
*q-DLP-19-2*	0.12	a2	2.75	1.35	0	2.25	0	1.69	*Glyma19g36280* (II)
*q-DLP-19-3*	0.11	a2	2.92	1.62	2.70	1.80	0	1.35	*Glyma19g41400* (III)
26		32					19 genes (3 I, 6 II, 4 III, 1 IV, 2 V, 3 VIII) 25 QTL-alleles (3 I, 9 II, 6 III, 1 IV, 2 V, 4 VIII)		

Note: SV: susceptible varieties; MRV: moderately resistant varieties; RV: resistant varieties. Non-shaded alleles are increased in MRV or RV compared to SV, while light-shaded alleles are decreased in MRV or RV compared to SV. The QTLs in boldface are LC QTLs. In total, 19 genes with 25 alleles were annotated from the 26 QTLs with 32 alleles in antixenosis changes from susceptible to moderate and resistant varieties.

## Data Availability

The raw data supporting the conclusions of this article will be made available by the corresponding author, without undue reservation.

## Supplementary Materials

The following supporting information can be downloaded at: https://www.mdpi.com/article/10.3390/ijms242216089/s1.
