# Supplementary material for: The Identification of a Quantative Trait Loci-Allele System of Antixenosis against the Common Cutworm (Spodoptera litura Fabricius) at the Seedling Stage in the Chinese Soybean Landrace Population
Source: Int J Mol Sci. 2023 Nov 8;24(22):16089. doi: 10.3390/ijms242216089 (PMC10671034; doi:10.3390/ijms242216089)
Supplement: Supplementary file 1 [file ijms-24-16089-s001.zip › ijms-2647351-supplementary.pdf]

Supplementary Materials

Supplementary Tables

Table S1 Joint ANOVA of DLP for antixenosis against CCW at seedling stage in CSLRP

Source	*DF*	*MS*	*F-value*
Accession	369	1327.0	6.3**
Env.	2	32033.0	152.2**
Block (Env.)	6	2347.5	18.7**
Env.×Accession	734	210.5	1.7**
Error	2177	125.4	

Note: ** represents significance at 0.01 probability level. *DF*: Degree of freedom; *MS*, Mean square.

Table S2 Multiple comparisons for antixenosis and antibiosis of highly resistant and susceptible accessions at seedling and adult stage

Variety	Antixenosis (%)		Antibiosis (mg)	
	DLP-S	DLP-A	Larva weight	Body weight increase
S01	20.8b	24.4ef	14.6gh	3.3efg
S02	18.9b	20.0ef	21.7efgh	1.7h
S03	19.8b	27.6def	19.3efgh	3.5de
S04	17.2b	39.8bcd	18.4efgh	2.9efg
S05	27.2b	30.8cde	14.9gh	2.4fgh
S06	23.1b	15.5f	15.6fgh	1.8h
S07	24.7b	21.5ef	23.9ef	2.4fgh
S08	26.4b	19.3ef	14.7gh	2.3gh
S09	27.8b	41.0bcd	23.5efg	4.5bcd
S10	24.2b	19.6ef	13.2h	3.4def
S11	74.9a	53.6ab	82.5ab	3.3efg
S12	64.2a	51.4ab	59.3d	3.9cde
S13	75.4a	40.4bcd	89.8a	5.1ab
S14	66.9a	43.9abc	81.1b	3.6de
S15	73.9a	49.5ab	88.6ab	5.8a
NN89-29	62.7a	54.7a	67.7c	4.8abc
Lamar	18.3b	17.4ef	25.2e	1.7h

Note: S01= AYHD; S02= LJHD; S03= AYLLZ; S04= AYXHD; S05= HZHD; S06= STXHD; S07= DJSSYH; S08= GZHD; S09= BLSYHH; S10= LWBMLYH; S11= BMD; S12= CDSLQ; S13= SRDQS; S14= LSZZHD; S15= FCDQD.

DLP-S is the DLP at seedling stage in 2017, 2018 and 2019, DLP-A is the DLP at adult stage in 2019. Larva weight is used as the indicator of antibiosis at seedling stage, while body weight increase is used as the indicator of antibiosis at adult stage. Values in a same column followed by different lowercase letter mean significant difference among varieties at $P \leq 0.05$ level.

Table S3 Correlation among traits of antixenosis and antibiosis at seedling and adult stage in CSLRP

Indicator	DLP-S	DLP-A	Larva weight	Consumed amount	Body weight increase
DLP-A	0.82**				
Larva weight	0.97**	0,69**			
Consumed amount	0.85**	0.72**	0.87**		
Body weight increase	0.71**	0.75**	0.70**	0.80**	
Excrement amount	0.69**	0.75**	0.67**	0.72**	0.91**

Note: DLP-S is DLP at seedling stag. DLP-A represents DLP at adult stage. Larva weight is the indicator of antibiosis at seedling stage, while consumed amount, body weight increase and excrement amount are the indicators of antibiosis at adult stage. ** represents significance at 0.01 level.

Table S4 QTL-allele constitution of the fifteen selected accessions in CSLRP

QTL	R^2	High-resistant accession										High-susceptible accession				
		S01	**S02**	S03	S04	**S05**	**S06**	**S07**	**S08**	S09	S10	S11	S12	S13	S14	S15
q-DLP-01-1	0.08	0.7	0.7	-0.7	0.7	-0.7	0.7	0.7	-0.7	0.7	-0.7	0.7	0.7	-0.7	0.7	-0.7
q-DLP-01-2	0.07	-1.7	-1.7	-1.7	-1.7	-1.7	1.7	-1.7	-1.7	-1.7	-1.7	-1.7	-1.7	-1.7	-1.7	-1.7
q-DLP-01-3	0.15	-3.1	-3.1	-3.1	-3.1	-3.1	-3.1	-3.1	-3.1	-3.1	-3.1	-3.1	-3.1	-3.1	-3.1	-3.1
q-DLP-01-4	0.39	-0.3	-0.3	-1.6	-0.3	-0.3	-0.3	-1.6	-0.3	0.0	-0.3	-2.7	-1.6	0.0	-1.6	-1.6
q-DLP-01-6	0.62	3.5	3.8	3.8	3.8	3.5	3.5	3.8	3.5	3.8	3.8	3.8	3.8	3.8	3.5	3.5
q-DLP-01-7	0.19	7.0	7.0	7.0	7.0	7.0	7.0	7.0	7.0	7.0	7.0	7.0	7.0	7.0	7.0	7.0
q-DLP-02-1	0.17	4.8	1.6	-0.8	-0.3	-0.8	-0.8	-0.8	-0.8	-0.8	-2.2	-2.2	4.8	-2.2	-0.8	-0.8
q-DLP-02-2	0.14	0.7	0.7	-2.3	0.7	0.7	0.7	0.7	0.7	0.7	0.7	-2.4	0.7	0.7	0.7	-2.4
q-DLP-02-3	0.57	2.1	-2.1	-2.1	-2.1	-2.1	-2.1	-2.1	-2.1	-2.1	-2.1	-2.1	-2.1	-2.1	-2.1	-2.1
q-DLP-02-4	0.14	2.4	2.4	2.4	2.4	2.4	2.4	2.4	2.4	2.4	2.4	2.4	2.4	2.4	2.4	2.4
q-DLP-02-5	0.71	0.4	0.4	-1.1	-3.3	0.4	-3.3	-3.3	0.4	0.4	-1.1	0.4	-1.1	-3.3	0.4	0.4
q-DLP-02-7	0.15	-4.9	-4.9	-4.9	-4.9	-4.9	-4.9	-4.9	-4.9	-4.9	-4.9	-4.9	4.9	-4.9	-4.9	-4.9
q-DLP-03-1	0.27	0.8	-3.9	3.0	3.0	3.0	-3.9	-3.9	0.8	3.0	3.0	3.0	0.8	0.8	3.0	3.0
q-DLP-03-2	0.15	-1.6	-1.6	-1.6	-1.6	-1.6	-1.6	-1.6	-1.6	-1.6	-1.6	-1.6	-1.6	-1.6	-1.6	-1.6
q-DLP-04-1	0.09	-2.7	-2.7	-2.7	-2.7	-2.7	-2.7	-2.7	-2.7	-2.7	-2.7	-2.7	-2.7	-2.7	-2.7	-2.7
q-DLP-06-1	0.47	-1.7	-0.8	2.5	2.5	2.5	-0.8	2.5	2.5	2.5	-0.8	2.5	2.5	2.5	-0.8	-0.8
q-DLP-06-2	0.07	-1.0	-1.0	-1.0	-1.0	1.0	-1.0	-1.0	1.0	-1.0	-1.0	-1.0	-1.0	-1.0	-1.0	-1.0
q-DLP-06-3	0.26	-7.5	-7.5	-7.5	-7.5	-7.5	-7.5	-7.5	-7.5	-7.5	-7.5	-7.5	-7.5	-7.5	-7.5	-7.5
q-DLP-06-5	0.28	-2.9	-2.9	-2.9	-2.9	-2.9	-2.9	-2.9	-2.9	-2.9	-2.9	-2.9	-2.9	-2.9	-2.9	-2.9
q-DLP-06-6	0.75	4.1	4.1	0.9	4.1	0.9	-0.9	-0.9	-0.5	0.9	0.9	0.9	0.9	0.9	0.9	0.9
q-DLP-06-7	0.54	-1.8	-1.8	1.8	-1.8	-1.8	1.8	1.8	-1.8	1.8	1.8	1.8	1.8	1.8	1.8	1.8
q-DLP-07-1	0.15	-1.7	-1.7	-1.7	-1.7	-1.7	-1.7	-1.7	-1.7	-1.7	-1.7	-1.7	-1.7	-1.7	-1.7	-1.7
q-DLP-08-1	0.19	-1.7	-0.3	-0.3	2.0	-0.3	-0.3	-0.3	2.0	-1.7	-0.3	-0.3	2.0	-0.3	-0.3	-0.3
q-DLP-08-2	2.16	-9.0	-3.9	-3.9	-9.0	-6.2	-6.2	-3.9	-6.7	-9.0	-6.2	-6.2	-6.7	-3.9	-3.9	-6.2
q-DLP-08-3	0.86	3.1	3.1	8.1	3.1	2.8	2.8	2.8	2.8	2.8	8.1	8.1	8.1	3.1	2.8	3.1
q-DLP-08-4	0.06	2.8	-2.8	2.8	2.8	2.8	2.8	2.8	2.8	2.8	2.8	2.8	2.8	2.8	2.8	2.8
q-DLP-08-6	0.68	-2.6	-2.6	2.6	2.6	2.6	-2.6	-2.6	-2.6	-2.6	-2.6	2.6	2.6	2.6	2.6	-2.6
q-DLP-08-7	1.55	11.0	11.0	11.0	11.0	11.0	-11.0	11.0	11.0	11.0	11.0	11.0	11.0	11.0	11.0	11.0
q-DLP-08-8	0.66	-2.5	-2.5	-2.5	-2.5	0.5	-2.5	-2.5	0.5	-2.5	-0.7	-2.5	-2.5	0.5	0.5	4.8
q-DLP-10-1	0.76	-1.4	3.1	3.1	3.1	3.1	3.1	3.1	3.1	3.1	3.1	3.1	3.1	3.1	3.1	3.1
q-DLP-10-3	0.87	-7.4	-7.4	-7.4	-7.4	-7.4	-7.4	-7.4	-7.4	-7.4	-7.4	-7.4	-7.4	-7.4	-7.4	7.4
q-DLP-10-4	1.46	2.0	-6.5	-2.0	2.0	-2.0	0.2	-2.0	-2.0	-2.0	-2.0	-2.0	-2.0	0.2	-2.0	-2.0
q-DLP-10-5	0.19	-4.6	4.6	4.6	4.6	4.6	4.6	4.6	4.6	4.6	4.6	4.6	4.6	-4.6	4.6	4.6
q-DLP-11-2	0.23	1.6	1.6	0.7	0.7	0.7	1.6	0.5	0.7	0.5	0.7	0.7	0.7	0.7	0.5	0.7
q-DLP-12-1	0.34	-4.4	4.4	4.4	-4.4	4.4	4.4	4.4	4.4	4.4	4.4	4.4	4.4	4.4	4.4	4.4
q-DLP-12-2	0.14	-2.2	-2.2	-2.2	-2.2	-2.2	-2.2	-2.2	-2.2	-2.2	-2.2	-2.2	-2.2	-2.2	2.2	2.2
q-DLP-12-3	0.09	-1.3	-1.3	-1.3	-1.3	-1.3	-1.3	-1.3	-1.3	-1.3	-1.3	-1.3	-1.3	-1.3	-1.3	-1.3
q-DLP-12-4	5.16	8.4	8.4	-0.7	-0.7	-0.7	8.4	-9.5	-0.7	8.4	-0.7	8.4	8.4	8.4	8.4	8.4
q-DLP-12-6	0.38	-7.1	-7.1	-7.1	-7.1	-7.1	-7.1	-7.1	-7.1	-7.1	-7.1	-7.1	-7.1	-7.1	-7.1	-7.1
q-DLP-12-7	0.30	-6.4	-6.4	-6.4	-6.4	-6.4	-6.4	-6.4	-6.4	-6.4	-6.4	-6.4	-6.4	6.4	-6.4	-6.4
q-DLP-12-8	0.12	-2.5	-2.5	-2.5	-2.5	-2.5	-2.5	-2.5	-2.5	-2.5	-2.5	-2.5	-2.5	-2.5	-2.5	-2.5
q-DLP-13-1	0.83	8.5	8.5	8.5	-8.5	8.5	8.5	8.5	8.5	8.5	8.5	8.5	8.5	8.5	8.5	8.5
q-DLP-13-2	0.14	-2.3	2.3	2.3	2.3	2.3	2.3	2.3	2.3	-2.3	2.3	2.3	2.3	2.3	2.3	2.3
q-DLP-13-3	1.88	2.5	-6.5	-4.7	2.5	-4.7	4.8	-4.7	3.0	-6.3	-4.7	-4.7	-4.7	-4.7	-2.4	-0.4
q-DLP-13-4	0.35	1.6	-1.6	1.6	-1.6	1.6	1.6	1.6	1.6	1.6	1.6	1.6	1.6	1.6	1.6	1.6
q-DLP-13-6	0.32	6.3	6.3	-6.3	6.3	6.3	6.3	6.3	6.3	6.3	6.3	6.3	-6.3	6.3	6.3	6.3
q-DLP-13-8	1.00	-2.1	3.3	-2.1	-2.1	-1.2	-2.1	3.3	-2.1	-1.2	-1.2	-2.1	3.3	3.3	3.3	-2.1
q-DLP-14-1	1.40	3.0	3.0	-0.9	3.0	3.4	3.4	3.0	-0.9	3.0	-0.9	-0.9	-0.9	-0.9	3.0	3.0
q-DLP-14-2	0.09	-2.8	-2.8	-2.8	-2.8	-2.8	-2.8	-2.8	-2.8	-2.8	-2.8	-2.8	-2.8	-2.8	-2.8	-2.8
q-DLP-14-3	0.17	2.7	2.7	2.7	2.7	-2.7	2.7	2.7	2.7	2.7	2.7	2.7	2.7	2.7	2.7	2.7
q-DLP-15-1	2.35	2.2	-2.2	-2.2	2.2	-2.2	-2.2	-2.2	-2.2	-2.2	-2.2	2.2	2.2	2.2	2.2	-2.2
q-DLP-15-2	3.08	-2.9	-2.9	-2.9	-2.9	-2.9	2.8	-2.9	-2.9	2.8	-2.9	2.8	-2.9	2.8	2.8	-2.9
q-DLP-15-3	0.21	3.0	-3.0	3.0	3.0	3.0	3.0	3.0	3.0	-3.0	3.0	3.0	3.0	3.0	3.0	3.0
q-DLP-17-1	1.91	-7.4	-7.4	-7.4	-7.4	-7.4	-7.4	-7.4	-7.4	-7.4	-7.4	17.6	-7.4	-1.7	-7.4	-1.7

q-DLP-17-2	0.06	-2.4	-2.4	-2.4	-2.4	-2.4	-2.4	-2.4	-2.4	-2.4	-2.4	-2.4	-2.4	-2.4	-2.4	-2.4
q-DLP-18-2	0.10	1.1	1.1	1.1	1.1	1.1	-1.1	1.1	1.1	-1.1	1.1	1.1	1.1	1.1	1.1	1.1
q-DLP-18-3	0.38	-7.9	-7.9	-7.9	-7.9	-7.9	-7.9	-7.9	-7.9	-7.9	-7.9	-7.9	-7.9	-7.9	-7.9	-7.9
q-DLP-18-4	2.44	-1.1	-1.1	-1.1	-1.1	-1.1	-1.0	-1.1	-1.1	-1.1	-1.1	-10.6	-1.1	-1.0	3.0	-1.1
q-DLP-18-5	0.20	4.9	4.9	4.9	4.9	4.9	4.9	4.9	4.9	4.9	4.9	4.9	4.9	4.9	4.9	4.9
q-DLP-18-6	0.14	1.4	1.4	-1.4	1.4	1.4	1.4	1.4	1.4	1.4	1.4	1.4	1.4	1.4	1.4	1.4
q-DLP-18-7	0.08	-0.5	-0.5	0.5	-0.5	0.5	-0.5	-0.5	-0.5	-0.5	-0.5	0.5	0.5	-0.5	-0.5	-0.5
q-DLP-19-1	1.07	0.9	-0.6	0.9	0.9	0.9	0.9	-0.6	0.9	0.9	-0.6	0.9	-0.6	-0.6	-7.5	0.9
q-DLP-19-2	0.12	-2.8	-2.8	-2.8	-2.8	-2.8	-2.8	-2.8	-2.8	-2.8	-2.8	-2.8	-2.8	-2.8	-2.8	-2.8
q-DLP-19-3	0.11	-2.9	-2.9	-2.9	-2.9	-2.9	-2.9	-2.9	-2.9	-2.9	-2.9	-2.9	-2.9	-2.9	-2.9	-2.9
q-DLP-20-1	0.10	-2.4	-2.4	-2.4	-2.4	-2.4	-2.4	-2.4	-2.4	-2.4	-2.4	-2.4	-2.4	-2.4	-2.4	-2.4
q-DLP-20-2	0.88	2.8	-3.9	-3.9	2.8	-3.9	-3.9	1.1	-3.9	-3.9	-3.9	1.1	-3.9	1.1	1.1	-3.9
DLP (%)		20.8	18.9	19.8	17.2	27.2	23.1	24.7	26.4	27.8	24.2	74.9	63.2	75.4	66.9	73.9
Sum of effects		-25.1	-42.0	-41.7	-34.3	-27.5	-38.1	-40.9	-27.4	-31.8	-34.3	11.2	-4.4	9.0	7.9	7.2
Negative allele no.		37	42	42	37	37	39	40	38	38	43	33	34	33	31	37
Positive allele no.		29	24	24	29	29	27	26	28	28	23	33	32	33	35	29

Note: The accessions in boldface are better than Lamar in antixenosis at seedling stage. The QTLs in boldface are LC-QTLs. The QTL marked with colors are QTLs changed from susceptible to moderate and resistant varieties (Table7), 11 QTLs without change among accessions are marked in green while 15 QTLs with change among accessions are marked in yellow~~ish~~. Among the 66 QTLs, 17 QTLs without change among the fifteen selected accessions while 49 QTLs with changes among the fifteen selected accessions.

Table S5 The allele changes of 66 main-effect QTLs in CSLRP

QTL	a1	a2	a3	a4	a5	a6	a7	a8	a9	a10	a11
q-DLP-01-1											
q-DLP-01-2											
q-DLP-01-3											
q-DLP-01-4											
q-DLP-01-6											
q-DLP-01-7	M,R										
q-DLP-02-1				M,R							
q-DLP-02-2											
q-DLP-02-3											
q-DLP-02-4	M										
q-DLP-02-5			M,R								
q-DLP-02-7		r									
q-DLP-03-1											
q-DLP-03-2											
q-DLP-04-1		r									
q-DLP-06-1											
q-DLP-06-2											
q-DLP-06-3		r									
q-DLP-06-5											
q-DLP-06-6	M,R	r					r				
q-DLP-06-7											
q-DLP-07-1											
q-DLP-08-1											
q-DLP-08-2											
q-DLP-08-3											
q-DLP-08-4	M,R										
q-DLP-08-6											
q-DLP-08-7	M,R										
q-DLP-08-8					r						
q-DLP-10-1	M,R	M,R									
q-DLP-10-3		r									
q-DLP-10-4	M,R					M,R					
q-DLP-10-5											
q-DLP-11-2					r						
q-DLP-12-1											
q-DLP-12-2											
q-DLP-12-3											
q-DLP-12-4											
q-DLP-12-6		r									
q-DLP-12-7		m									
q-DLP-12-8		r									
q-DLP-13-1	M,R										
q-DLP-13-2											
q-DLP-13-3	M,R									r	M,R
q-DLP-13-4											
q-DLP-13-6											
q-DLP-13-8											
q-DLP-14-1							M,R				
q-DLP-14-2											
q-DLP-14-3											
q-DLP-15-1											
q-DLP-15-2											
q-DLP-15-3											
q-DLP-17-1											
q-DLP-17-2		r									
q-DLP-18-2											
q-DLP-18-3		r									
q-DLP-18-4						M,R					
q-DLP-18-5											
q-DLP-18-6											
q-DLP-18-7											
q-DLP-19-1											
q-DLP-19-2		M									
q-DLP-19-3		r									
q-DLP-20-1											
q-DLP-20-2											

Note: a1–a11 are the possible alleles of each QTL, arranged in a rising order according to their effect value. RV: resistant varieties; MRV: moderately resistant varieties; SV: susceptible varieties. All the white background cells represent alleles with negative effect and all the grey background cells represent alleles with positive effect, and the cells without letters represent the alleles shared in MRV, RV (vs. SV). The cells with lowercase “m” “r” are alleles decreased in MRV, RV (vs. SV), respectively. The uppercase of “M” “R” in cells means the alleles increased in MRV, RV (vs. SV), respectively.
